# Triple florigenic signaling in rice promotes stem elongation via transcriptional de‐repression

**DOI:** 10.1111/tpj.71050

**Published:** 2026-07-19

**Authors:** Giulio Vicentini, Greta Bertagnon, Fabio Dobetti, Francesca Giaume, Francesca Cazzaniga, Alessio Baldini, Giulia Ave Bono, Jan Maika, Selina Sterup Moore, Alessandro Bignardi, Rüdiger Simon, Giorgio Perrella, Keisuke Nagai, Motoyuki Ashikari, Fabio Fornara, Vittoria Brambilla

**Affiliations:** ^1^ Department of Agricultural and Environmental Sciences University of Milan Via Celoria 2 20133 Milan Italy; ^2^ Department of Biosciences University of Milan Via Celoria 26 20133 Milan Italy; ^3^ Department of Biology and Biotechnology “C. Darwin” La Sapienza University of Rome Via dei Sardi 70 Rome Italy; ^4^ Institute for Developmental Genetics, Heinrich Heine University Düsseldorf North Rhine Westphalia Germany; ^5^ Bioscience and Biotechnology Center of Nagoya University Furo‐Cho, Chikusa Nagoya Aichi 464‐8602 Japan

**Keywords:** rice, stem elongation, floral transition, florigens, transcriptional reprogramming, chromatin remodelling

## Abstract

The beginning of the reproductive phase of cereals is marked by inflorescence formation and stem elongation. In rice, florigenic proteins encoded by *HEADING DATE 3a* (*Hd3a*) and *RICE FLOWERING LOCUS T 1* (*RFT1*) coordinate both developmental transitions by inducing expression of floral identity genes at the apical meristem and establishing stem competence to respond to gibberellins. Stem elongation is repressed during the vegetative phase by the activity of *PREMATURE INTERNODE ELONGATION 1* (*PINE1*), and the florigens reduce its transcription, thus relieving growth repression. Here, we first resolve the relationships occurring between regulatory factors at the shoot apical meristem and underlying stem tissues in controlling reproductive phase transitions. We next show that the PINE1 protein forms higher order complexes with TOPLESS‐related and HISTONE DEACETYLASE proteins and is required to repress a set of target genes by reducing histone acetylation levels and increasing chromatin accessibility. We propose that PINE1 controls chromatin accessibility through histone deacetylation, preventing stem growth, and that florigenic proteins act upstream of growth‐promoting signals.

## Significance Statement

Rice florigenic proteins Hd3a, RFT1, and FT‐L1 coordinate reproductive transition with stem elongation through repression of PINE1 expression, independently of floral MADS box proteins, relieving repression of internode growth. PINE1 represses growth by transcriptionally repressing target genes by interacting with TOPLESS‐related and HISTONE DEACETYLASE proteins. PINE1 is a chromatin‐based regulator linking florigen signaling to stem growth.

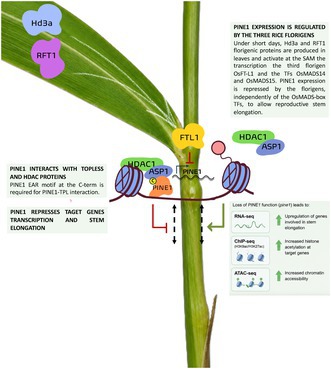

Flowering in rice is regulated by florigenic signaling that coordinates inflorescence development and stem elongation; during vegetative growth, stem elongation is repressed by PINE1, whose expression is rapidly downregulated at flowering. We show that PINE1 is directly repressed by each of the three rice florigens, but not by their earliest shoot apical meristem targets, and propose that PINE1 functions as a chromatin‐mediated transcriptional repressor.

## INTRODUCTION

As sessile organisms, plants have evolved a vast repertoire of environmental responses to adapt growth and physiology to changing conditions. The reproductive phase is particularly sensitive to environmental signals because the propagation of the species depends upon its correct completion. At the molecular level, sensor proteins relay external cues into gene regulatory networks ultimately responsible for modifying developmental programs.

In rice, the onset of flowering is accelerated by exposure to short days (SD), when day length falls below a critical threshold (Vicentini et al., 2023). These conditions promote transcription of *HEADING DATE 3a* (*Hd3a*) and *RICE FLOWERING LOCUS T 1* (*RFT1*) in the leaves. Their cognate proteins are long‐distance systemic signals that transmit a floral stimulus to the shoot apical meristem (SAM) and are referred to as florigens (Komiya et al., 2008; Tamaki et al., 2007). At the apex, the florigens induce transcription of *FLOWERING LOCUS T‐LIKE 1* (*FT‐L1*), encoding a florigen‐like protein required to promote flowering synergistically with Hd3a and RFT1, and to establish proper panicle architecture (Giaume et al., 2023). Florigens form higher order complexes, referred to as florigen activation complexes (FACs), that include members of the 14‐3‐3 protein family, and bZIP transcription factors that allow sequence‐specific DNA binding (Cerise et al., 2021; Taoka et al., 2011). The early targets of florigenic signaling include genes belonging to the MADS box family of transcription factors (Kobayashi et al., 2012), as well as other regulatory genes (Gómez‐Ariza et al., 2019; Mineri et al., 2023). However, the arrangement of these factors in gene networks at the SAM is still unclear.

During inflorescence development, the internodes, which are short and compressed during the vegetative phase, start to elongate, following a stereotypical pattern driven by the activation of intercalary meristems (Hoshikawa, 1989; Nagai et al., 2025). Usually, only four to five internodes below the SAM elongate, starting from the lowest. An acropetal gradient ultimately leads to elongation of the uppermost internode that sustains the inflorescence. Stem elongation begins when Hd3a and RFT1 reduce expression of the C2H2 transcription factor *PREMATURE INTERNODE ELONGATION 1* (*PINE1*), allowing the intercalary meristems to respond to gibberellins (GA), which promote cell division and expansion (Gómez‐Ariza et al., 2019; Kaneko et al., 2003). Loss‐of‐function *pine1* mutants elongate the internodes constitutively and uncouple flowering from stem growth. *PINE1* is also known as *DECELERATOR OF INTERNODE ELONGATION 1* (*DEC1*), underlying a QTL controlling stem growth in deepwater rice (Nagai et al., 2014, 2020). Deepwater rice comprises varieties characterized by fast internode elongation upon submergence and cultivated in flood‐prone areas of Southeast Asia. The deepwater trait allows some leaves to be kept above the water surface, providing oxygen to submerged tissues. Under submergence, ethylene is produced, and its accumulation induces transcription of a deepwater‐specific haplotype of *SEMI DWARF 1* (*SD1*), encoding a GA20 oxidase (OsGA20ox2) responsible for biosynthesis of bioactive GA (Kuroha et al., 2018). Unlike conventional varieties, deepwater rice harbors variants of *PINE1*/*DEC1* that are transcriptionally silenced by GA or deepwater treatments. Therefore, *PINE1*/*DEC1*'s role as a suppressor of elongation is correlated to its expression. High transcript abundance is associated with reduced stem growth and, when *PINE1*/*DEC1* is overexpressed, it causes severe plant size reduction. Conversely, conditions that downregulate *PINE1*/*DEC1* expression are permissive for stem elongation. These examples also indicate that *PINE1*/*DEC1* (hereafter *PINE1*) is central to unrelated processes whose common component is stem growth (Nagai & Ashikari, 2023).

In this study, we analyzed the molecular function of PINE1 and determined its role as a transcriptional repressor.

## RESULTS

### Florigens are required for *PINE1* downregulation and stem elongation

**Figure 1:**
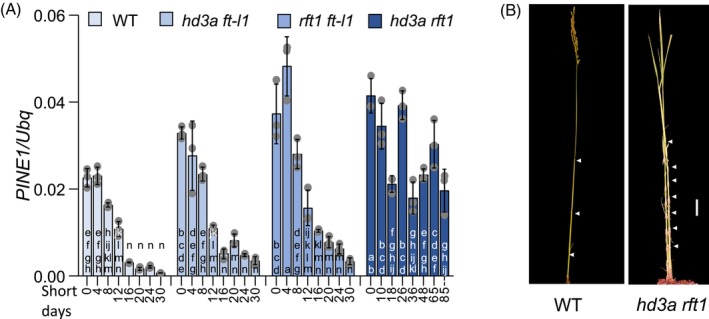
Florigen‐dependent and independent stem elongation. (A) Quantification of *PINE1* transcription in florigens double‐mutant combinations. Plants were grown under LD for 6 weeks and harvested at the indicated day after shifting to SD. Samples were collected at *Zeitgeber* (ZT) 0. Histograms show the mean ± standard deviation of three technical replicates. Letters indicate statistical significance determined at *P* < 0.05 by one‐way anova. A biologically independent experiment gave similar results. SD, short day. (B) Internodes elongation pattern in *hd3a rft1* double‐mutant stems compared with WT. The WT plants grown for 8 weeks under LDs and then 12 weeks under SD carry fully ripened panicles and show typical maximum elongation of the higher internodes, while *hd3a rft1* double mutants of the same age show reduced internode elongation and an atypical elongation pattern with longer lower internodes. Leaves were removed to expose nodes that are indicated by white arrowheads. Scale bar = 5 cm. WT, wild‐type.

Panicle development is accompanied by the onset of *OsMADS* transcription, most prominently *OsMADS14* and *OsMADS15* (Kobayashi et al., 2012), whereas internode elongation is caused by reduction of *PINE1* expression (Gómez‐Ariza et al., 2019; Nagai et al., 2020). The leaf‐derived florigens Hd3a and RFT1 are necessary and sufficient to induce expression of several *OsMADS* genes and sufficient to reduce *PINE1* transcription (Gómez‐Ariza et al., 2019; Mineri et al., 2023). FT‐L1 functions as a third florigen and is specifically expressed in the SAM upon the arrival of Hd3a or RFT1 proteins. Whether *FT‐L1* also promotes internode elongation independently from H3da and RFT1 is unknown. Equally unknown is the relative hierarchy of these regulators and their targets at the SAM. We quantified transcription of *PINE1* in stem samples that included the SAM (Figure S1A), in genotypes having reduced (*hd3a ft‐l1* and *rft1 ft‐l1*) or abolished (*hd3a rft1*) florigenic activity (Figure 1A). Since *FT‐L1* expression relies on the presence of Hd3a and/or RFT1 in the SAM, in *hd3a rft1* double‐mutant *FTL‐1* is not expressed in this tissue. When wild‐type (WT) plants grown under LD were shifted to SD to induce *Hd3a* and *RFT1* transcription, expression of *PINE1* decreased rapidly, becoming hardly detectable after 30 SD. In *hd3a ft‐l1* and *rft1 ft‐l1* the pattern of decrease was similar to the WT at the beginning of floral induction, but, while by Day 30 *PINE1* expression was not detectable in the WT, some *PINE1* mRNA was still present in the double florigenic mutants at this timepoint. Therefore, residual activity of RFT1 and Hd3a in *hd3a ft‐l1* and *rft1 ft‐l1*, respectively, was sufficient to reduce *PINE1* transcript abundance, but not to WT levels. Conversely, in *hd3a rft1* double mutants, expression of *PINE1* did not decrease to the same extent, even after 85 SD, when the experiment was concluded (Figure 1A). Since *hd3a rft1* double mutants fail to induce *FT‐L1* transcription, effectively behaving like a triple mutant (Giaume et al., 2023), florigenic activities are necessary to reduce *PINE1* expression.

The *hd3a rft1* double mutant could grow vegetatively for more than 3 years without flowering. We observed that after several months of vegetative growth, the mutant elongated the internodes. However, the pattern was different from the WT because ll elongating internodes reached similar lengths, and the beginning of elongation was dependent upon age rather than a specific developmental transition (Figure 1B).

### Position of *PINE1* in the florigens regulatory network

**Figure 2:**
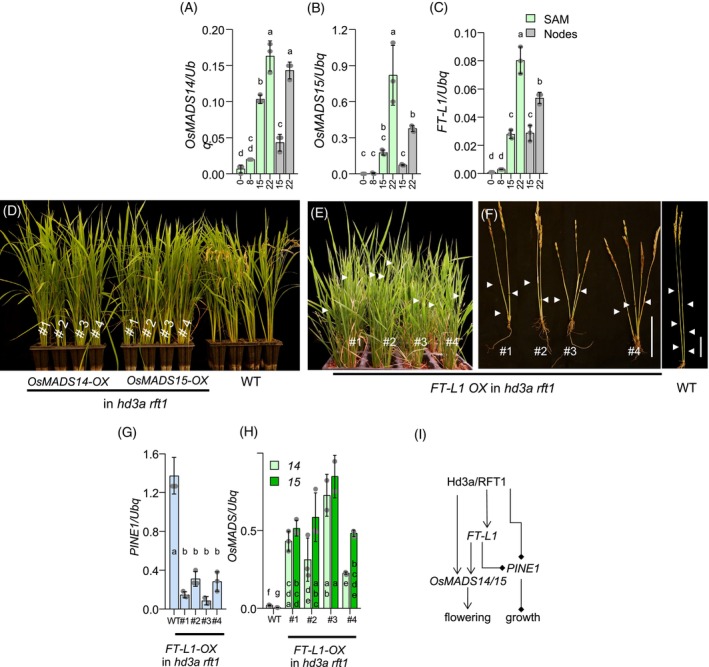
Position of *PINE1* in the florigens regulatory network. (A–C) Quantification of *OsMADS14* (A), *OsMADS15* (B), and *FT‐L1* (C) transcription in SAMs and nodes of Nipponbare. Plants were grown under LD for 6 weeks and harvested at the indicated day after shifting them to SD. Samples were collected at ZT 0. SD, short day. (D) Gross morphology of *pOSH1:OsMADS14* and *pACT:OsMADS15 hd3a rft1* plants, compared with the wt. (E) *pOSH1:FT‐L1 hd3a rft1* at the flowering stage. Numbers indicate independent transgenic lines. White arrowheads indicate the panicles. (F) Stems of mature *pOSH1:FT‐L1 hd3a rft1*. Leaves were removed. White arrowheads indicate the nodes. Scale bars, 10 cm. (G, H) Quantification of *PINE1*, *OsMADS14*, and *OsMADS15* transcription in the genotypes shown in (D) to (F). Plants were grown under LD for 6 weeks and harvested at ZT 0. (I) Model showing the regulatory circuitry dependent upon the florigens. Arrows and diamonds indicate transcriptional activation and repression, respectively. Quantification of transcripts was done using technical triplicates, and each data point is shown on the bars. Letters on the bars indicate statistical significance, determined at *P* < 0.05 by one‐way anova. Each quantification experiment was repeated at least twice with biologically independent samples, giving similar results.

To position PINE1 in the florigen induction network, we analyzed its relationship with the three genes activated at the SAM during the earliest stages of floral induction, including *OsMADS14*, *OsMADS15*, and *FT‐L1*. First, we quantified their transcriptional response to SD in the SAM and nodes containing intercalary meristems, where *PINE1* is strongly expressed before floral induction. We observed that similar dynamics of increased expression were evident both in the SAM as well as in stem portions containing the intercalary meristems, albeit to a lesser extent (Figure 2A–C; Figure S1B). These results indicate that *PINE1* could function downstream of *Hd3a*/*RFT1* as well as *OsMADS14*/*15* and *FT‐L1*. To uncouple florigenic signaling from the expression of MADS box genes, we overexpressed *OsMADS14* under the control of the *ORYZA SATIVA HOMEOBOX 1* promoter (*pOSH1*), which is expressed in the SAM and underlying stem (Tsuda et al., 2011), and *OsMADS15* under the control of the ubiquitous *ACTIN* promoter (*pACT*) (Brambilla et al., 2017) in the *hd3a rft1* double mutant. Both promoters guaranteed a similar level of expression that was comparable to that observed in plants that are irreversibly committed to flowering by the florigens (Figure S2A,B). Despite the high, near‐physiological level of expression of *OsMADS14* or *OsMADS15*, neither *pOSH1:OsMADS14 hd3a rft1* nor *pACT:OsMADS15 hd3a rft1* plants flowered or elongated the internodes, indicating that Hd3a and RFT1 are essential for flowering and internode elongation even if *OsMADS14*/*15* are expressed (Figure 2D). We next resolved the regulatory relationships between *Hd3a*/*RFT1* and their downstream direct target *FT‐L1* in the control of *PINE1* transcription. To this end, we expressed *FT‐L1* under the control of the *pOSH1* promoter in the *hd3a rft1* double mutant. The *pOSH1:FT‐L1 hd3a rft1* transgenics flowered early under LD, producing a terminal small panicle bearing few florets (Figure 2E,F). Some plants already flowered in the rooting medium (Figure S2C,D). The reproductive organs were fertile, and plants could produce seeds, but all florets converted the sterile glumes into bracts (Figure S2E). Most importantly, the plants elongated the internodes at the base of the inflorescence and had reduced expression of *PINE1* compared with WT plants grown under the same conditions (Figure 2F,G). Consistently with the ability to develop an inflorescence, expression of *OsMADS14* and *OsMADS15* was induced in the *pOSH1:FT‐L1 hd3a rft1* SAM under LD (Figure 2H). This result resembles that obtained by overexpressing *Hd3a* in the *ft‐l1* mutant background (Giaume et al., 2023), suggesting that expression of any florigenic gene is independently sufficient to trigger flowering, as well as internode elongation.

Taken together, these data indicate that *PINE1* functions downstream of *Hd3a*, *RFT1*, and *FT‐L1* and in a pathway that does not depend on *OsMADS14* and *OsMADS15* expression (Figure 2I; Figure S8).

### PINE1 EAR motifs are evolutionarily conserved

**Figure 3:**
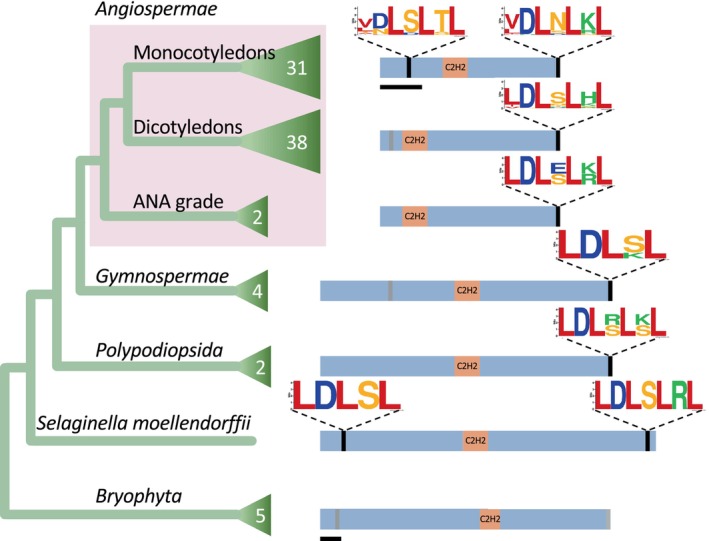
Evolutionarily conservation of PINE1 EAR motifs. Sequence homology between PINE1 homologs from early land plants, ferns, and Gymnosperms is limited to the C2H2 zinc finger domain, but N‐ and C‐terminal EAR motifs were identified in all lineages. Numbers within triangles indicate the number of species used to infer consensus sequences. A schematic diagram of proteins representative of each clade is shown, highlighting the position of the C2H2 domain. EAR motifs are indicated by black vertical lines when present in all species of the clade, and by gray vertical lines when only some species of the clade harbor it. Horizontal black lines indicate 50 amino acids. ANA, Amborellales Nympheales Austrobaileales.

PINE1 is a transcription factor harboring two ethylene‐responsive element binding factor‐associated amphiphilic repression (EAR) motifs, located at the N‐ and C‐termini of the protein (hereafter EARN— consensus LxLxLxL— and EARC— consensus LxLxL—, respectively) (Figure S3A). We retrieved amino acid sequences of the closest PINE1 homologs from all Embryophytes clades and observed that EARN and EARC could be identified in all lineages even when a relatively low number of sequences were identified (Figure 3A). While the EARN motif was not consistently present in all species from the same clade and was absent from fern sequences, the EARC motif was identified in all species from *Selaginella* to Angiosperms. In dicotyledons, the EARN motif was identified in species belonging to Solanales and Fagales. Only in monocotyledons both EARN and EARC motifs were consistently identified in all species. These data suggest that EARN and EARC were present in homologs of PINE1 in early land plants and the EARN motif was most subject to loss during evolution. However, the consistent presence of EARN in monocots suggests a functional role.

### PINE1 interacts with a TOPLESS and a HISTONE DEACETYLASE proteins

**Figure 4:**
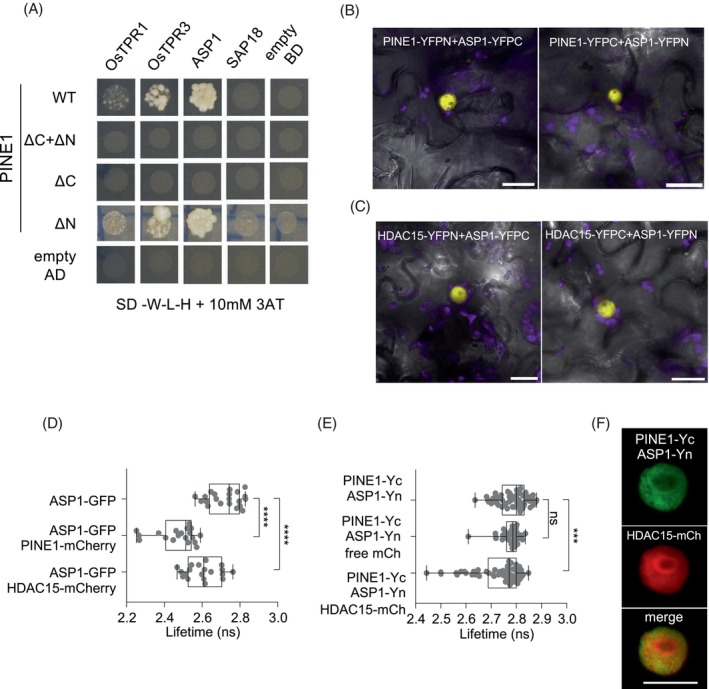
PINE1 interacts with a TOPLESS and a HISTONE DEACETYLASE protein. (A) Yeast‐two hybrid showing interaction between OsTPR1, OsTPR3, ASP1, and SAP18 fused to the Gal4‐binding domain (BD) and PINE1 versions harboring mutations in either (ΔC or ΔN) or both (ΔC + ΔN) EAR motifs. Diploid yeast was grown on selective medium lacking tryptophan, leucine, and histidine (SD‐W, ‐L, ‐H) and supplemented with 10 mM 3‐amino‐1,2,4‐triazole (3AT). SD, short day. (B, C) Bimolecular fluorescence complementation (BiFC) experiments performed in tobacco, between the indicated protein pairs, fused to either the N‐terminal (YFPN) or C‐terminal (YFPC) of YFP. YFP fluorescence is shown in yellow, while chloroplasts autofluorescence is shown in purple. Scale bar: 20 μm. (D, E) Box plots showing FRET‐FLIM analysis of the interaction between ASP1‐PINE1 and ASP1‐HDAC15 (D) and between the PINE1‐ASP1 dimer and HDAC15 (E). Interaction strength was measured by reduction in lifetime (ns) of GFP/YFP in the presence of mCherry (mCh). The boxes show the median and the 25th–75th percentile range; the whiskers indicate the full data range. Each dot represents a measurement of a nucleus. These experiments were repeated five times. Asterisks indicate statistical significance based on unpaired, two‐tailed Student's *t*‐test. ***, *P* < 0.001; ****, *P* < 0.0001; ns, non‐significant. (F) Example of a nucleus imaged for FRET‐FLIM analysis. The top and middle pictures show the YFP and mCherry signals, respectively, while the bottom image shows the merge. Scale bar 10 μm.

EAR motifs recruit TOPLESS‐like co‐repressors, including TOPLESS‐RELATED 1 (OsTPR1), OsTPR3, ABERRANT SPIKELET AND PANICLE 1 (ASP1), and SIN3‐ASSOCIATED POLYPEPTIDE 18 (SAP18), in turn mediating interaction with histone deacetylases (HDACs) (Geng et al., 2020; Ke et al., 2015; Yoshida et al., 2012). We cloned the coding sequences of OsTPR1, OsTPR3, ASP1, and SAP18, and tested their interaction with PINE1. OsTPR3 and ASP1 could interact with PINE1, whereas OsTPR1 and SAP18 could not. Truncation of the EARC (from wt LNLKL* to ΔC LN*) (Figure S3A) abolished the interaction. Conversely, mutagenesis of the EARN (from wt LSLTLGL to ΔN ISVTLGI) did not modify the interaction patterns in yeast, suggesting that only the EARC is essential for interaction between PINE1, OsTPR3, and ASP1 (Figure 4A). When tested for interaction strength, the PINE1‐ASP1 interaction was slightly stronger than that with OsTPR3, promoting better yeast growth (Figure S3B). The interaction between PINE1 and ASP1 was then confirmed by bimolecular fluorescence complementation (BiFC) (Figure 4B; Figure S3C) and by FRET/FLIM (Figure 4D) in tobacco leaves. We then used ASP1 to determine if it could recruit HDACs. We cloned six rice HDACs chosen for their expression at the shoot apex (HDT2, HDAC9, HDAC1, HDAC15, HADC5, and HDA6) and attempted their expression in yeast, but this impaired growth. Therefore, we performed a preliminary FRAP assay (data not shown) to test interaction with ASP1. Following these tests, HDAC15 showed a clear interaction, and we proceeded with FRET‐FLIM analysis. By FRET‐FLIM, HDAC15 could interact with ASP1, and the interaction took place in the nucleus (Figure 4C,D). Finally, we combined split YFP and FRET‐FLIM to assess the formation of a trimer. PINE1 and ASP1 were fused to the C‐ and N‐terminal portions of YFP, respectively, and co‐infiltrated with HDAC15‐mCherry in tobacco leaves. The lifetime of the reconstituted YFP decreased when HDAC15‐mCherry was present, but not when an empty vector carrying mCherry only was infiltrated (Figure 4E). PINE1, ASP1, and HDAC15 colocalized in the nucleus (Figure 4F). Collectively, these data indicate that PINE1 can form a complex comprising ASP1 and an HDAC, and that HDAC15 can be one of the HDACs present in this complex. This is likely to be a transcriptional repression complex and PINE1 EARC motif seems necessary for the PINE1‐ASP1 interaction in yeast.

### Both EAR motifs are important for PINE1 function *in vivo*

**Figure 5:**
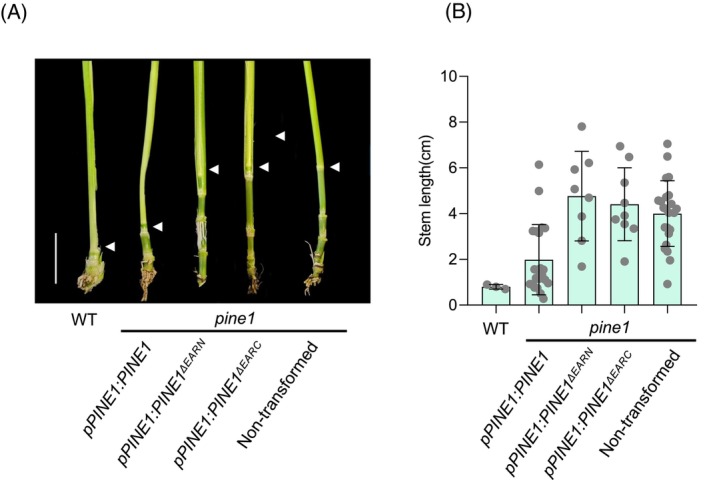
Deletion of PINE1 EAR motifs impairs PINE1 function. (A) Stems of WT and *pine1* mutant plants complemented with PINE1 full length (*PINE1*) or missing EARN (*PINE1* ^ΔEARN^) or EARC (*PINE1* ^ΔEARC^), expressed under the control of *PINE1* promoter *(pPINE1)*. WT, wild‐type. (B) Stem length of vegetative WT and *pine1* mutants complemented with full‐length PINE1 or versions missing an EAR motif. The bars show the mean ± standard deviation. Each dot in the transgenic lines represents a measurement from an independent transformant. Scale bar: 1 cm.

Since evolutionary conservation studies (Figure 3A) and published structures (Ke et al., 2015) suggest the importance of both EAR motifs in the interaction with three alpha helices of ASP1, we tested their impact *in vivo*. To this end, we complemented *pine1* mutant plants either with a WT cDNA clone of *PINE1* expressed under the control of ≈2 kb upstream promoter sequence (*pPINE1:PINE1 pine1*) or with *PINE1* mutated in the EARN or EARC motifs (Figure S3A). Expression of the constructs was tested in the different transgenic lines (Figure S3C). While *pPINE1:PINE1* plants could largely complement premature internode elongation (Figure 5A,B), *pPINE1:PINE1* ^*ΔEARC*^ or *pPINE1:PINE1* ^*ΔEARN*^ harboring the same EAR mutations used in yeast‐two‐hybrid experiments could not repress *pine1* internode elongation during vegetative growth. These data support that, while the EARN is evolutionarily less conserved and its deletion does not abolish interaction with ASP1 in yeast, both EAR motifs seem essential for PINE1 function *in vivo*.

### Transcriptional activation via histone hyperacetylation in *pine1*

As molecular and genetic data suggested the formation of a transcriptional repressor complex where PINE1 binds TOPLESS co‐repressors and HDACs, we indirectly verified this hypothesis by performing RNA‐seq, ChIP‐seq using antibodies against acetylated H3K9K14 and ATAC‐seq analyses on WT and *pine1* 3 mm shoot apices including the meristems. Although it is unlikely that PINE1‐ASP1‐HDAC15 is the sole complex that can deacetylate H3K9K14 in stems, we still expect an increase in acetylation in *pine1* mutant, specifically on the complex's targets.

**Figure 6:**
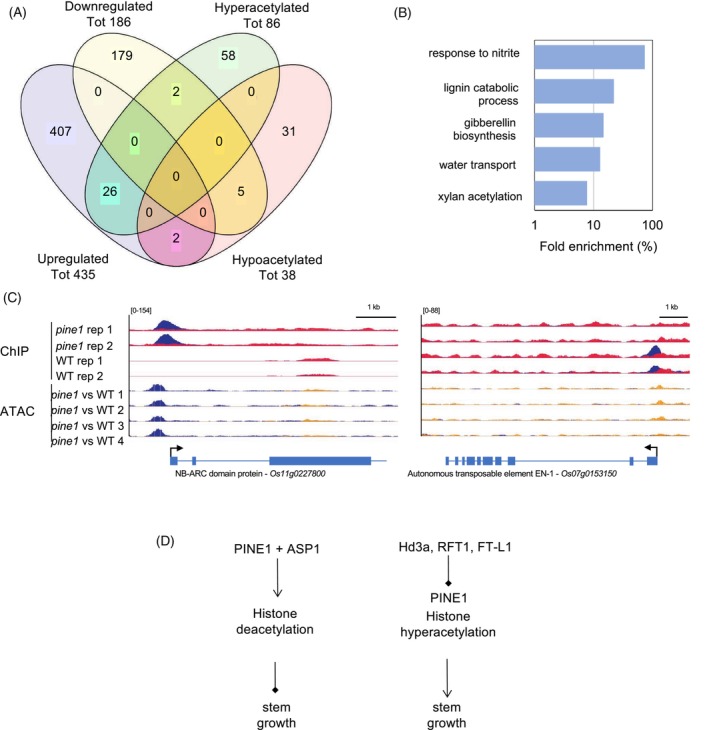
Identification of PINE1 targets by RNA‐, ChIP‐, and ATAC‐sequencing. (A) Venn diagram showing genes differentially expressed (filtered for FDR <0.05 and log_2_FC >|1|) and differentially acetylated in the *pine1* mutant, based on RNA‐seq and ChIP‐seq. (B) Gene ontology of genes differentially expressed in *pine1* mutant versus WT. (C) Examples of acetylation and DNA accessibility patterns in genes upregulated (left) or downregulated (right) in the *pine1* mutant. The y‐axis represents the normalized read coverage. Red and blue signals indicate the input and immunoprecipitated samples of ChIP‐seq, respectively. Blue and orange indicate the read coverage of *pine1* and WT of ATAC‐seq, respectively. Two biological replicates for ChIP‐seq and four for ATAC‐seq are indicated. Gene structures are represented at the bottom of the graphs. Boxes represent exons, lines represent introns. A black arrow indicates the direction of transcription. The scale (1 kb) is shown on the top right of each graph. (D) Model of PINE1 molecular function.

RNA‐seq identified 621 differentially expressed genes in the mutant, 70% (435) of which were repressed by PINE1, consistent with its primary function as a transcriptional repressor (Figure 6A; Figure S4; Table S1). Ontological categories enriched among differentially expressed genes included terms related to cell wall remodeling, water transport, and GA metabolism (Figure 6B), in agreement with PINE1's effect on cell elongation and GA sensitivity already reported in the mutant (Gómez‐Ariza et al., 2019; Nagai et al., 2020). Next, we used antibodies recognizing acetylated H3K9K14 and quantified changes in chromatin states by ChIP‐seq. We identified 132 differentially acetylated chromatin regions (corresponding to 123 genes), 70% of which were hyperacetylated in *pine1* mutants (Figure 6A; Figure S5; Table SS2). We intersected RNA‐seq and ChIP‐seq data and found 26 genes being both upregulated and hyperacetylated in *pine1* mutants, whereas 5 were downregulated and hypoacetylated (Figure 6A; Table S4). This subset of targets included the most upregulated genes, and acetylation was observed mostly around the transcriptional start site or in the gene body (Figure 6C; Table S4).

To examine the relationship between PINE1, acetylation levels, and chromatin accessibility, we performed ATAC‐seq to compare *pine1* mutants and the WT, sampling the same tissues used for RNA‐ and ChIP‐seq. We identified 1018 differentially accessible regions, 634 of which were more accessible in the mutant (Figure S6; Table S3). We next examined the genes resulting from the overlap between RNA‐ and ChIP‐seq and observed that almost all hyperacetylated and upregulated genes (23/26) were also more accessible in the *pine1* mutant (Table S4). The DNA regions corresponding to increased histone acetylation corresponded to regions of increased accessibility, usually at the beginning of the transcriptional start site (Figure 6C).

Almost all these candidates have no molecular function or biological process predictions. We independently validated three target genes chosen among the overlap between the three datasets (Figure 3A; Table S5) by performing ChIP‐qPCR and quantitative reverse transcriptase‐polymerase chain reaction (qRT‐PCR) on newly collected stem samples (Figure S7).

We propose a working model whereby PINE1 recruits histone deacetylation complexes to loci promoting activity of the intercalary meristems, maintaining them inactive. Conversely, reducing *PINE1* expression as a consequence of floral induction and Hd3a, RFT1, and OsFT‐L1 expression relaxes chromatin compaction, activating targets that ultimately allow stem growth (Figure 6D; Figure S8).

## DISCUSSION

### PINE1 is a general regulator of stem elongation

The study of stem elongation in rice has benefited from the use of distinct models, including the SAM undergoing transition from vegetative to reproductive growth and deepwater varieties whose stems elongate during submergence. Regulatory factors have emerged that include *PINE1* and *ACCELERATOR OF INTERNODE ELONGATION 1* (*ACE1*), having repressive and promoting activities on internode elongation, respectively. Additionally, extensive genetic data have clarified that *PINE1* and *ACE1* act in independent pathways and are sufficient to modulate GA responses. However, while deepwater varieties harbor functional alleles of *ACE1* and maintain *PINE1* transcription low, which enhance GA responses, conventional paddy rice expresses functional *PINE1* and internodes are not responsive to GA treatments. Stems of paddy rice do not elongate in response to GA even though SLENDER RICE 1 (SLR1), the sole DELLA protein in rice and repressor of GA signaling, is degraded in upon GA treatments. Therefore, at the molecular level, the relationship between *PINE1*, GA signaling, and stem elongation is still not completely understood. In this study, we better resolved the position of *PINE1* in the regulatory network controlling the transition to flowering, showing that it acts in a branch that is downstream of *FT‐L1* but independent of *OsMADS14*/*15* activities. This position in the regulatory network has two major implications. First, *PINE1* temporally acts after the florigens and *FT‐L1*, suggesting that its expression may be dependent upon a FAC. In FACs, DNA target specificity is provided by a dimer of bZIP transcription factors that mediate binding to specific G‐box motifs in the promoter of regulated genes. Florigen activities are mediated by the assembly of FACs not only to induce the floral transition (Taoka et al., 2011) but also to regulate other developmental processes in rice, such as formation of axillary buds (Tsuji et al., 2015) and branching of the inflorescence (Giaume et al., 2023). Extending the concept to other species, FACs have been shown to regulate diverse processes, including shoot determinacy and crop productivity in tomato (Park et al., 2014), tuber formation in potato (Teo et al., 2017) and seasonal growth in aspen (Tylewicz et al., 2015). Therefore, FACs could also operate in intercalary meristems to promote stem elongation. The bZIP candidates that could take part in such complexes include the characterized OsFD1‐4, OsFD7, Hd3a BINDING FACTOR 1 (HBF1), and HBF2 (Brambilla et al., 2017; Cerise et al., 2021; Kaur et al., 2021; Taoka et al., 2011; Tsuji et al., 2015). However, none of their corresponding mutants has been reported to affect internode elongation, suggesting redundancy or the existence of other factors. A thorough genetic analysis of higher order mutants is required to corroborate the idea of FACs controlling stem elongation, as well as detailed expression analyses of complex components in the stem.

A second aspect relates to spatial regulation of internode elongation. Detailed morphological analysis in C9285, a deepwater variety, indicated that elongation occurs in two steps, including a primary mild elongation of the foot region and a secondary extensive elongation caused by intercalary meristems at the base of the internodes (Nagai et al., 2025). While the morphological changes taking place in the stem of paddy rice might slightly differ, a conserved feature in the two model systems is the onset of elongation at a distance of several nodes from the SAM. This implies that in paddy rice, the activities of florigens and/or FT‐L1 in the control of *PINE1* expression extend beyond the SAM. Since Hd3a and RFT1 are systemic long‐distance signals produced in leaves, it is plausible to postulate that they could reach the base of the internodes to promote establishment or proliferation of the intercalary meristems. This hypothesis is corroborated by expression data indicating transcription of florigen targets in nodal tissues (this study), and by the existence of extensive vascular anastomoses at the nodes that could facilitate florigens' movement to the intercalary meristems (Nagai et al., 2025). Alternatively, upon reaching the SAM, the florigens could activate a second diffusible signal establishing an elongation gradient along the stem. The second signal could be a protein, a hormone, or a ligand. Further studies are necessary to test these hypotheses.

Stem elongation can also occur independently of the stimuli that induce *PINE1* downregulation. We observed that in the *hd3a rft1* non‐flowering double mutant, which does not repress *PINE1* even after an extended period, the internodes eventually elongate. We hypothesize that the internodes could acquire competence to elongate upon aging, with a mechanism independent of PINE1. The distinct nature of such a mechanism is evident also from the elongation pattern. During the transition to flowering, only the youngest, uppermost internodes acquire the capacity to elongate and grow with a characteristic pattern whereby the internodes elongate more if closer to the apex (Gómez‐Ariza et al., 2019; Nagai et al., 2020, 2025). On the contrary, in age‐related stem growth, all internodes elongate to a similar extent. Thus, the spatial and temporal dynamics of *PINE1* repression remain a key determinant distinguishing florigen‐dependent from age‐dependent elongation.

### Molecular mechanism of PINE1 activity

We have shown that PINE1 acts as a transcriptional repressor, based on several pieces of evidence. PINE1 has two EAR motifs, with the EARC mediating the interaction with TPR3 and ASP1 co‐repressors. The EAR motif is the most common transcriptional repression motif found in plants, with consensus sequence LxLxL or DLNx(x)P. Mixed types are also relatively common, and the EARC of PINE1 has a DLNLKL‐type motif. The functional significance of mixed motifs is not understood, although it has been speculated that they could confer additional flexibility in protein–protein interactions, expanding the range of possible partners (Chow et al., 2023). Also, the presence of double EAR motifs indicates the potential to establish more contacts with interactors. Our complementation assays using PINE1 variants with mutated EARs indicated that both are necessary for protein function, but it remains unclear the molecular role of the EARN because it is not required for interaction with TPRs, ASP1, or SAP18. Studies in Arabidopsis have indicated that a second EAR motif of AUXIN/INDOLE ACETIC ACID 7 (IAA7) has a minor role compared with the first. While mutations in the first EAR abolish protein function, mutations in the second do not (Lee et al., 2016). Yet, the second EAR of IAA7 could interact with a subset of interactors recognized by the first EAR, and mutations in both EARs had additive effects on some phenotypes, indicating that the second EAR has functions similar to the first, even though with minor roles. This example highlights flexibility in the use of dual EARs by transcriptional repressors. We can only speculate about the function of the EARN. It could either help stabilize the EARC‐TPR or EARC‐ASP1 complex *in vivo*, or it may interact with additional factors that are necessary for PINE to fully exert its repressive function. A role in PINE1 protein stability cannot be excluded either.

Gene expression, chromatin acetylation, and accessibility data have shown that PINE1 can modify chromatin structure. PINE1 presence generally promotes histone deacetylation and increases heterochromatic content, while its absence allows new targets required for stem elongation to become transcriptionally accessible. This finding agrees with the fact that TPRs can recruit HDACs, with transcription factors mediating DNA‐binding specificity at target loci. Recruitment of HDACs by TPRs/EAR‐containing transcription factors complexes is necessary for targeted repression of genes regulated by the circadian clock (Wang et al., 2013), auxin (Plant et al., 2021), brassinosteroid (Oh et al., 2014), and ethylene (Deng et al., 2022) signaling. Yet, histone deacetylation is not the only mode of gene repression mediated by EAR domain transcription factors. In Arabidopsis, some of them recruit simultaneously both HDACs as well as POLYCOMB‐REPRESSIVE COMPLEX 2 (PRC2), promoting H3 lysine 27 (H3K27) trimethylation to repress gene expression (Baile et al., 2021). Therefore, EAR domain transcription factors act as hubs mediating complex chromatin rearrangements. PINE1 contributes to the maintenance of transcriptional repression across a broad set of genes, potentially through its incorporation into multiple repressive complexes. The identity of additional PINE1‐interacting proteins remains unknown; however, their identification may provide important insights into how PINE1 interfaces with hormone‐signaling pathways that coordinate growth and organ patterning.

## MATERIALS AND METHODS

### Plant material, growth conditions, and measurements

The variety Nipponbare was used in this study. The *pine1* mutant background has been described in (Gómez‐Ariza et al., 2019). Plants were grown under controlled LD (16‐h light/8‐h dark) or SD (10‐h light/14‐h dark) conditions with day and night temperatures set at 28°C and 24°C, respectively. Stem elongation of the *pine1* mutant in complementation assays was measured after 2 months of growth in LD condition. To evaluate statistical significance, unpaired one‐way anova analysis or Student's *t*‐test was performed. SAM, developing panicles, and nodes were sampled as depicted in Figure S1A using a surgical blade at ZT0. After sampling, they were quickly frozen in liquid nitrogen and stored at −80°C.

### RNA isolation and analysis of gene expression

Tissues from either SAM, developing panicles, or nodes were lysed using a Retsch Mixer Mill MM400. RNA was extracted using Nucleozol one phase RNA purification (Macherey‐Nagel, Düren, Germany) following the manufacturer protocol. DNAse treatment was performed using AMBION Turbo DNAse, and a standard precipitation was performed using ethanol and sodium acetate. cDNA synthesis was performed using ImProm‐II™ Reverse Transcriptase (Promega, Madison, WI, USA) following the producer instructions. qRT‐PCR was performed using 2× Maxima SYBR Green qPCR Master Mix (Thermo Scientific, Waltham, MA, USA). Measures were conducted in technical triplicates. Each experiment has been performed with at least biological duplicates, producing comparable results.

For RNA sequencing, Nipponbare WT and *pine1* 3 mm shoot apices including the meristems were sampled at 20 days after germination. About 8 SAMs per sample were taken, in triplicate. RNA was extracted using the Trizol reagent. Library preparation and sequencing was conducted by Novogene using Illumina 150 bp paired‐end reads. Fastq‐mcf was used to clip adapters and eliminate low‐quality/short reads. After trimming, at least 40 million reads were present in each sample. FastQC (http://www.bioinformatics.babraham.ac.uk/projects/fastqc/) was then used to assess reads quality. Gene annotation from IRGSP‐1.0 2020‐06‐03 and Os‐Nipponbare‐Reference‐IRGSP‐1.0 (https://rapdb.dna.affrc.go.jp/download/irgsp1.html) were used to map reads to Nipponbare genes using STAR. Read count was performed using htseq‐count (https://htseq.readthedocs.io/en/release_0.11.1/count.html), and differentially expressed genes were detected using R package DESEQ2 with a *P*‐value <0.0001. Raw data and processed data (differentially expressed gene list) are available at the Gene Expression Omnibus (GEO) with the series record number GSE267946. RNA data quality analyses are reported in Figure S4.

### Vector construction and rice transformation

*PINE1, OsTPR1, OsTPR3, ASP1, SAP18, HDT2 (Os01g0909100), HDAC9 (Os04g0409600), HDAC1 (Os06g0583400), HDAC15 (Os07g0164100), HDA5 (Os07g0602200)*, and *HDA6 (Os08g0344100)* were used for Yeast‐2‐Hybrid screenings. They were amplified from SAM cDNA and cloned in pDONR207 using Gateway™ BP Clonase™ II enzyme mix (Thermo Scientific). Finally, they were cloned using Gateway™ LR Clonase™ II enzyme mix in destination plasmids pGADT7‐GW and pGBKT7‐GW.

EAR motifs modifications were introduced by PCR in the *PINE1* coding sequence, by using a reverse primer introducing a premature STOP codon in the C‐terminal EAR motif or by overlapping PCR to modify the N‐terminal EAR motif. *PINE1, ASP1*, and *HDAC15* were amplified without STOP codon and Gateway™ cloned in pBatTL‐B‐s YFPc, pBatTL‐B‐sYFPn, pAbind‐mCherry, and pAbind‐GFP for BiFC and FRET‐FLIM. *OsMADS14* and *OsMADS15* were amplified and Gateway™ cloned in *pOSH1:GW* and *pACT:GW* (Brambilla et al., 2017), respectively. The construct *pOSH1:OsFT‐L1* was already described in (Giaume et al., 2023). The *pPINE1:PINE1* clones for complementation assays were amplified from gDNA, using the same methodology followed for Yeast 2‐Hybrid to modify the EAR motifs. Final products were cloned in pMPGWB401 (Xie et al., 2015). Primers and gRNA sequences are listed in Table S3.

For each rice transformation, about 100 Nipponbare seeds were used to induce callus formation. T‐plasmid containing the construct of interest was transformed via electroporation in *Agrobacterium tumefaciens* EHA105 cells and used to transform calli. The transformation process and media used are the same described by Hiei et al., with minor modifications (Hiei et al., 1994).

### Protein–protein interaction assays

Yeast transformation was carried out using PEG/lithium acetate as described in Agatep et al. (1998). pGADT7 and pGBKT7 plasmids were transformed in the AH109 and Y187 strains, respectively. Mating occurred overnight in YPAD + 10% PEG 3350. Mating occurrence was checked in SD‐W‐L plates. Interactions were checked in SD‐W‐L‐H + 2.5 mM 3AT, SD‐W‐L‐H + 5 mM 3AT, SD‐W‐L‐H + 10 mM 3AT, and SD‐W‐L‐H‐A. Each pair of interactors was tested also by swapping GAL4 activation and binding domains. The experiment was repeated three times, giving the same results.

For BiFC assays, *A. tumefaciens* EHA105 was transformed via electroporation with the plasmids of interest. A suspension of *A. tumefaciens* (OD = 0.4) carrying expression vectors of the two proteins to be tested, and the silencing suppressor p19. *Agrobacterium tumefaciens* was then infiltrated in leaves of *Nicotiana benthamiana* with a needleless syringe. After 2–5 days the agroinfiltrated part was isolated and screened for YFP signal on a Nikon A1 confocal microscope.

FRET‐FLIM on PINE1‐ASP1 and ASP1‐HDAC15 was carried out similarly to the BiFC. An OD of 0.5 was used for agroinfiltration. Plants were then incubated for 2–5 days. Transgene expression was induced by spraying a 20 μM solution of β‐estradiol on the leaves. After 16 h, GFP lifetime was measured without and with the acceptor mCherry on an average of 20 transformed cells using an SP8 DIVE FALCON spectral multiphoton FLIM microscope (Leica Microsystems, Wetzlar, Germany) and data were analyzed using LAS‐X FLIM/FCS module. For trimeric interactions PINE1‐ASP1‐HDAC15, a reconstituted YFP was used as donor and mCherry as acceptor. Free mCherry was used in combination with the YFP as negative control. Tobacco leaves were agroinfiltrated with an OD of 0.5 for each construct and plants were incubated for 3 days. Infected leaves were then sprayed with 20 μM solution of β‐estradiol to induce transgene expression. After 3 h, the YFP lifetime was measured without and with the acceptor mCherry. Fluorescence lifetime was measured at a Zeiss (Oberkochen, Germany) LSM 780 confocal microscope (40× water immersion objective, Zeiss C‐PlanApo, NA 1.2). For TCSPC a PicoQuant Hydra Harp 400 (PicoQuant, Berlin, Germany) was used. Photon counting was performed with a picosecond resolution. YFP was excited with a 485 nm (LDH‐D‐C‐485, 32 MHz, PicoQuant, Berlin, Germany) pulsed polarized laser. Laser power was adjusted to 1–2 μW. Light emitted from the sample was separated by a polarizing beam splitter before photons were selected with a band‐pass filter. For YFP, a 534/30 band‐pass filter was used, and for mCherry a 607/70 band‐pass filter. Photons were detected in both donor and acceptor channel simultaneously with Tau‐SPADs (PicoQuant, Berlin, Germany). Images were acquired at zoom 8 with a resolution of 256 × 256 pixel with a pixel size of 0.1 μm, and a pixel dwell time of 12.54 μs and laser repetition rate of 32 MHz. Photons were collected over 60 frames. The fluorescence decays of selected ROIs in the FLIM image were analyzed with the SymPhoTime FLIM analysis software (SymPhoTime 64, version 2.4). TCSPC bins of channel 1 and 2 (parallel and perpendicular light) were binned by 16 resulting in a bin width of 16 ps. Nuclei were selected by hand using the ROI tool. All samples were fitted with the FLIM analysis tool (Fitting model: *n*‐exponential reconvolution, model parameter *n* = 2).

### EAR domain conservation analysis

Sequences were retrieved from GenBank using PINE1 as query and aligned with MEGA (ver. 11.0.13). EAR motifs were identified manually, and logo plots computed using WebLogo (https://weblogo.berkeley.edu/logo.cgi).

### Chromatin immunoprecipitation

For ChIP, Nipponbare WT and *pine1* 3 mm shoot apices including the meristems were sampled at 20 days after germination. Chromatin immunoprecipitation was performed using 1–2 g of ground tissue powder as previously described with minor modifications (Perrella et al., 2024). For each experiment shoot meristems from WT and *pine1* mutant were used to extract chromatin. Bioruptor (Diagenode, Seraing (Liège), Belgium) was used to shear the chromatin using 40 cycles consisting each of 30 sec on and 30 sec off at high power. Anti‐H3K9K14 acetylation antibody (Diagenode pAb‐005‐050) was used to immunoprecipitate the chromatin combined with protein A Dynabeads (Thermofisher 10001‐D). Library preparation, sequencing and bioinformatic analysis has been performed by Biomarker technology (BMKGENE, Beijing, China). Sequencing was performed using Illumina 150 bp paired‐end reads. Trimmed reads were mapped to the rice genome (https://rapdb.dna.affrc.go.jp/download/irgsp1.html ver. 2023‐09‐07). The Integrative Genomics Viewer (Robinson et al., 2011) was used to visualize the distribution of mapped reads on the rice genome. MACS2 v2.2.7.1 (Zhang et al., 2008) was used for peak calling and ChIPseeker (Yu et al., 2015) was used for peak annotation. DESeq2 (Love et al., 2014) was then utilized for differential peak counts. ChIP‐seq data quality analyses are reported in Figure S5. All ChIP data are available at the GEO database with accession number GSE271920.

### ATAC‐seq

For ATAC‐seq, Nipponbare WT and *pine1* 3 mm shoot apices including the meristems were sampled at 20 days after germination. A total of eight samples were processed for generating 284.89 M raw reads. After quality control, 284.72 M clean reads were generated. Minimum 94.87% of clean reads achieved a quality score of Q30. Reads were mapped on the reference genome (https://rapdb.dna.affrc.go.jp/download/irgsp1.html) using Bowtie2.

ATAC‐seq data quality analyses are reported in Figure S6.

## AUTHOR CONTRIBUTIONS

VB, FF, MA, and GV conceptualized the project. VB, FF, MA, KN, RS, and GP developed methodology and provided supervision. GV, GB, FG, FC, JM, SSM, and AB conducted the experiments. VB acquired fundings and administered the project. FF, VB, and GV wrote the original manuscript draft, that was shared with and revised by all authors.

## CONFLICT OF INTEREST

None of the authors have a conflict of interest to disclose.

## Supporting information


**Figure S1.** Morphology of wild‐type stem and tissue sampled.
**Figure S2.** Quantification of *OsMADS14/15 OX* transgene overexpression and additional phenotypes of *OsFT‐L1 OX* lines.
**Figure S3.** PINE1 protein structure, interaction studies in yeast, negative controls of BiFC experiments, and expression of *PINE1* complementation lines.
**Figure S4.** RNA‐seq data quality.
**Figure S5.** ChIP‐seq data quality.
**Figure S6.** ATAC‐seq data quality.
**Figure S7.** Datasets validation.
**Figure S8.** Working model.

**Table S1.** List of genes differentially expressed in the wild‐type compared with the *pine1* mutant.

**Table S2.** List of differentially acetylated genomic regions in the *pine1* mutant compared with the wild‐type.

**Table S3.** List of differentially accessible genomic regions in the *pine1* mutant compared with the wild‐type.

**Table S4.** List of differentially expressed, acetylated, and accessible genomic regions in the *pine1* mutant compared with the wild‐type.

**Table S5.** List of primers and gRNAs used in this study.

## Data Availability

The data that support the findings of this study are openly available in GEO at https://www.ncbi.nlm.nih.gov/geo/query/acc.cgi?acc=GSE267946, reference number GSE267946; GSE271920.
