# Supplementary material for: Triple florigenic signaling in rice promotes stem elongation via transcriptional de‐repression
Source: Plant J. 2026 Jul 19;127(2):e71050. doi: 10.1111/tpj.71050 (PMC13381055; doi:10.1111/tpj.71050)
Supplement: Supplementary file 1 — Figure S1. Morphology of wild‐type stem and tissue sampled. Figure S2. Quantification of OsMADS14/15 OX transgene overexpression and additional phenotypes of OsFT‐L1 OX lines. Figure S3. PINE1 protein structure, interaction studies in yeast, negative controls of BiFC experiments, and expression of PINE1 complementation lines. Figure S4. RNA‐seq data quality. Figure S5. ChIP‐seq data quality. Figure S6. ATAC‐seq data quality. Figure S7. Datasets validation. Figure S8. Working model. [file TPJ-127-0-s002.pdf]

SUPPLEMENTARY FIGURES

FIGURE S1

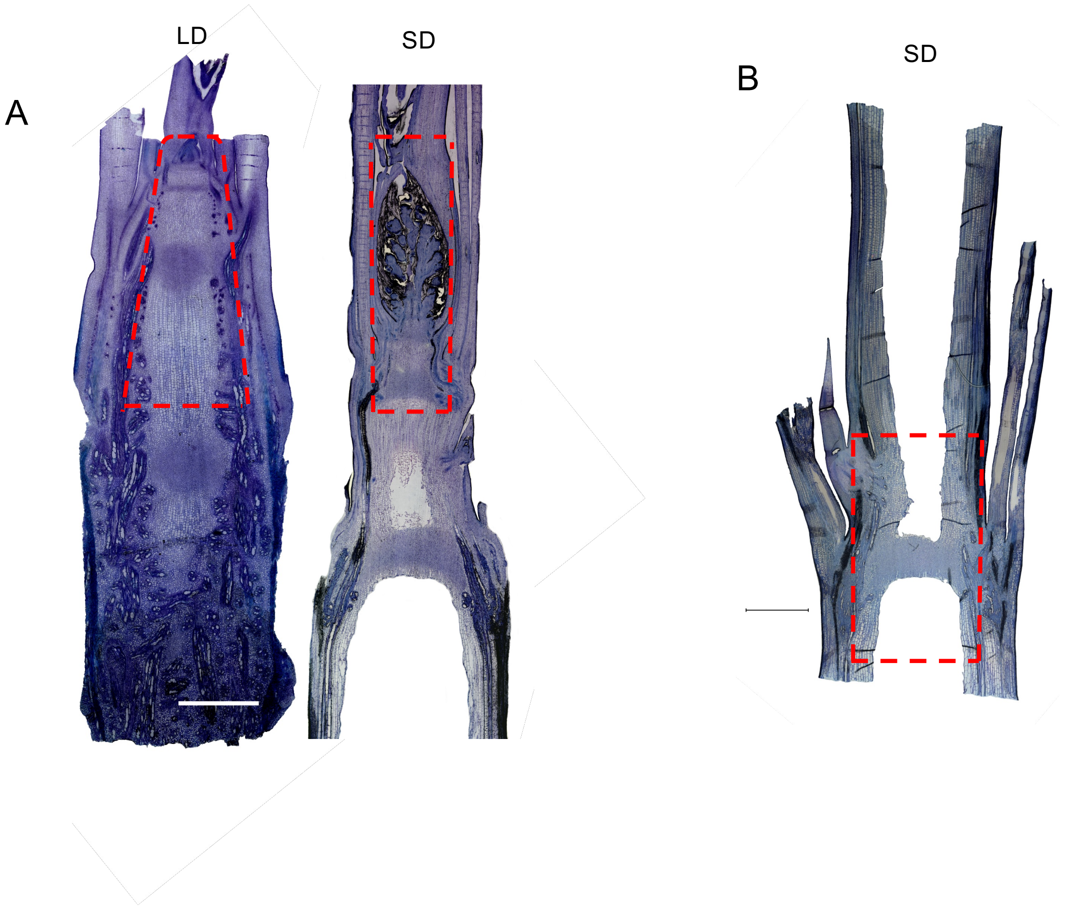

Fig S1. Morphology of wild type stems and tissue sampled. (a) Longitudinal sections of WT rice SAMs during LD growth (left) and after exposure to 21 SD. The red dotted line delimits the tissue sampled for qRT-PCR and RNA-seq experiments. Scale bar = 1 mm. (b) Longitudinal section of a WT stem portion comprising of a node and intercalary meristem. The red dotted line delimits the tissue sampled for qRT-PCRs shown in Fig 1b-d. Scale bar = 1 mm.

FIGURE S2

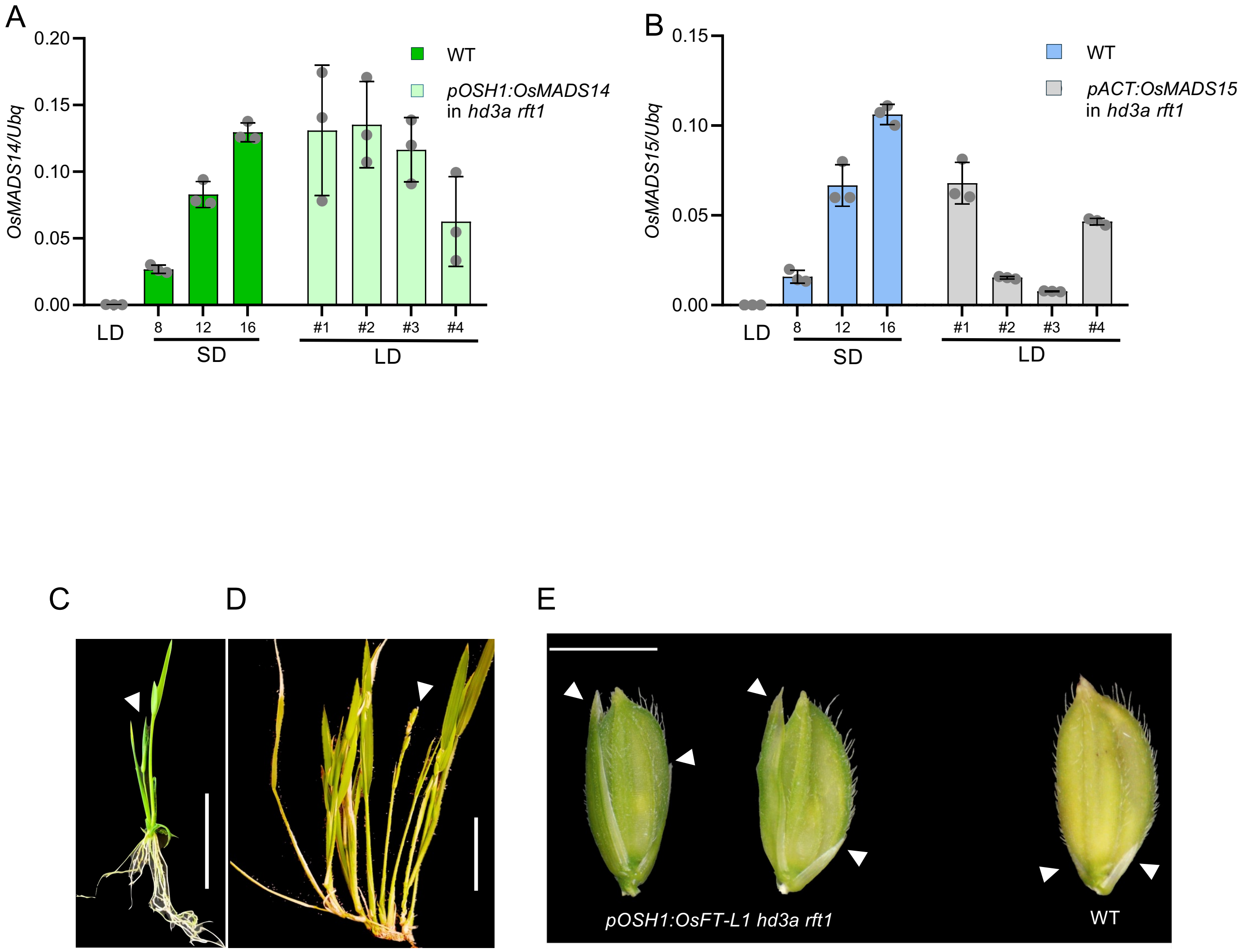

Fig S2. Quantification of *OsMADS14/15 OX* transgene overexpression and additional phenotypes of *OsFT-L1 OX* lines . Quantification of *OsMADS14* (a) and *OsMADS15* (b) expression by qRT-PCR in WT plants grown under LD or after exposure to 8, 12 or 16 SD and in four independent *hd3a rft1* mutant lines transformed with *pOSH1:OsMADS14 or pACT:OsMADS15,* respectively. (c-d) Two *pOSH1:OsFT-L1 hd3a rft1* plants flowering in rooting medium. The white arrowheads indicate the panicles. Scale bars: 5cm. (d) *pOSH1:OsFT-L1 hd3a rft1* plants (left) carry spikelets with elongated sterile glumes that cover either the palea or both palea and lemma. In WT plants, sterile glumes do not elongate (right). Arrowheads indicate the sterile glumes. Scale bar = 3mm.

FIGURE S3

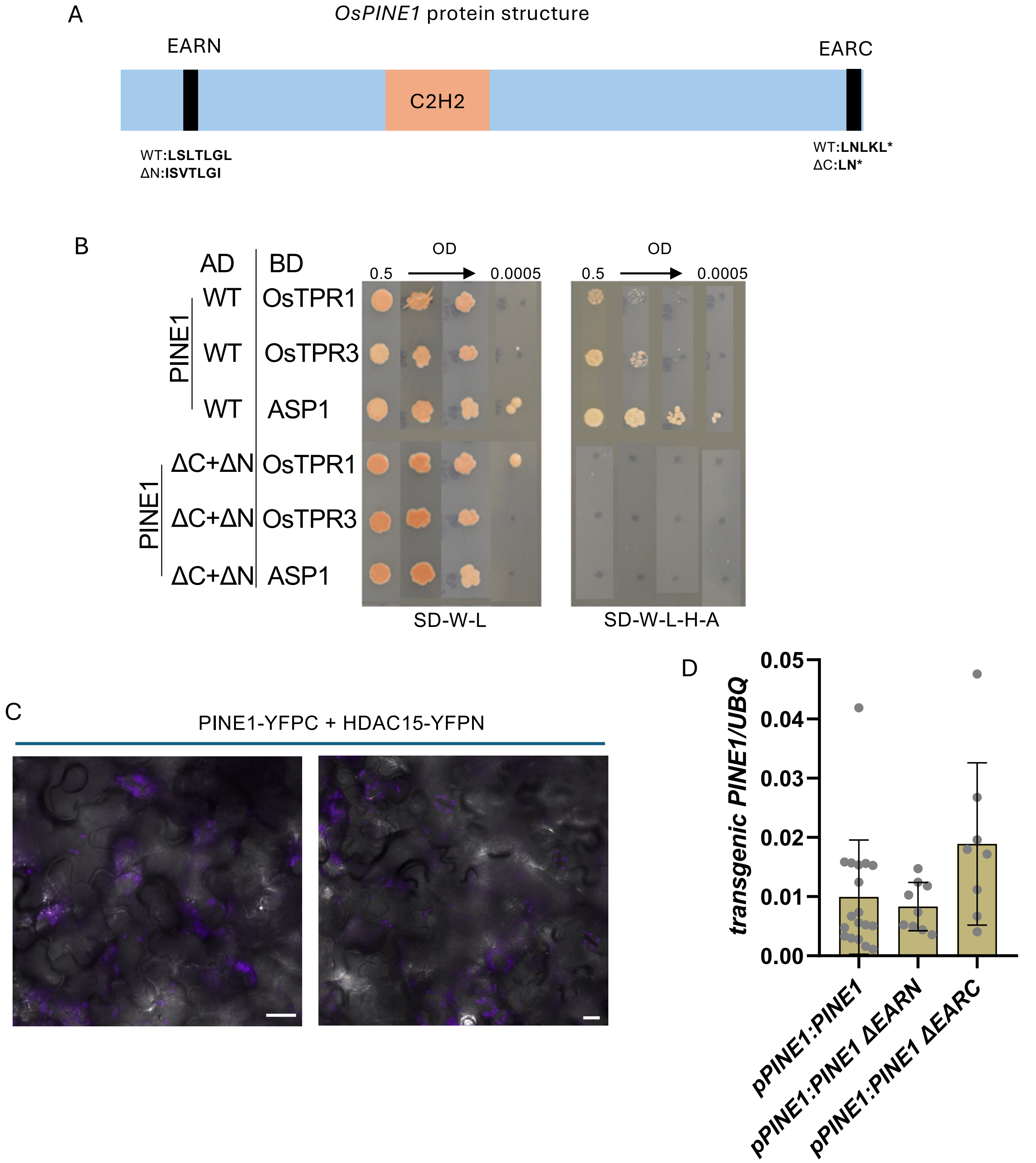

Fig S3. PINE1 protein structure, interaction studies in yeast ,BIFC negative controls and construct expression in *PINE1ΔN* and *PINE1ΔC* complementation lines. (a) PINE1 protein structure with position of EARN and EARC motifs: WT sequences and ΔN and ΔC mutations used in yeast two hybrid clones and complementation lines. (b) Yeast-two-hybrid between wild type and ΔC+ΔN PINE1 variants, and TPR1, TPR3 and ASP1. In this experiment, 10-fold serial dilutions of mated yeast (ranging from OD 0.5 to OD 0.0005) were plated on SD -L-W and SD -L-W-H-A media, to assess mating and interaction strength, respectively. Note that a weak interaction between PINE1 and TPR1 was evident in this experiment; the interaction between PINE1 and ASP1 was strongest. AD and BD indicate the activation and binding domains of Gal4, respectively. (c) Negative control for BiFC experiments: no fluorescence is observed when direct interaction between PINE1 and HDAC15 is tested. Scale bar: 20μm. (d) Espression of transgenic *PINE1* in complementation lines. Histograms show the mean ± standard deviation of three technical replicates. Each dot represents the mean of the measurement from an independent transformant.

FIGURE S4

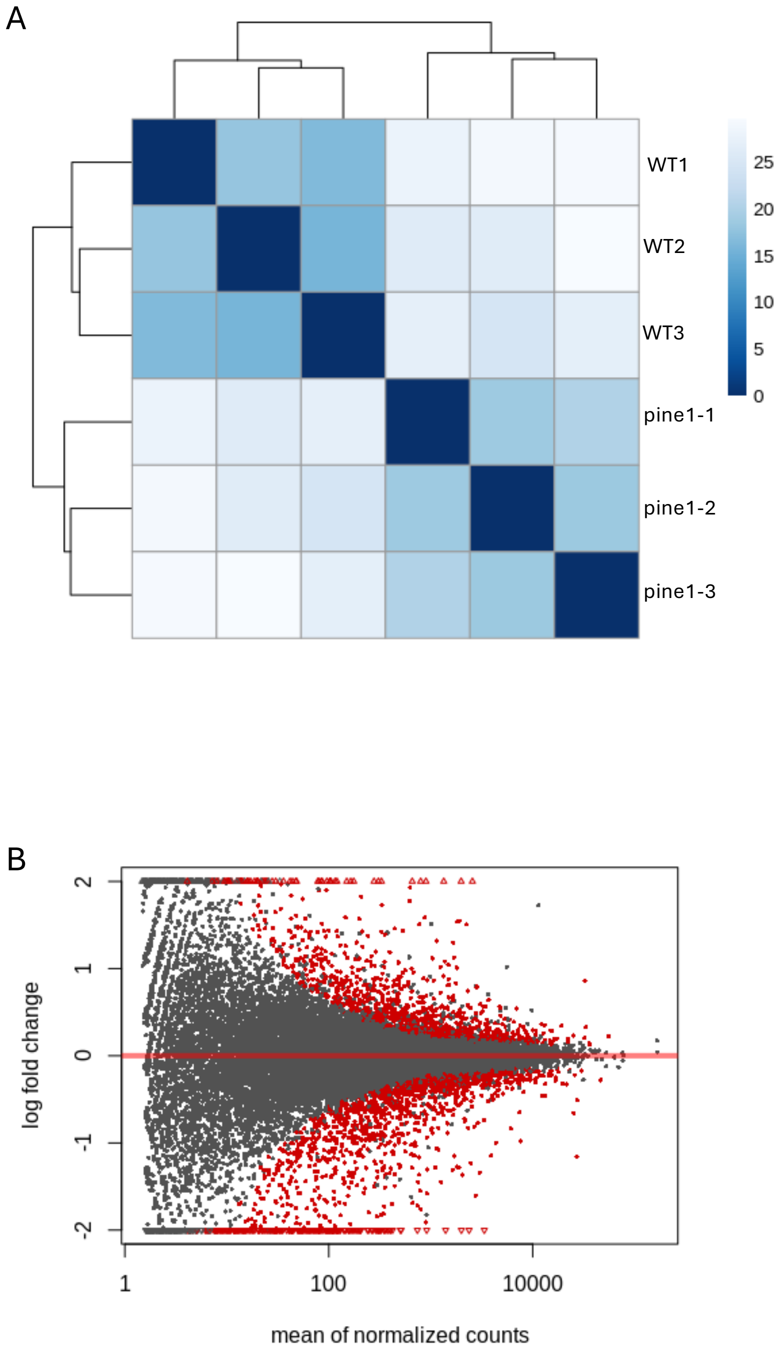

Fig.S4. RNA-Seq data quality (a) Sample to sample distances between WT and *pine1* replicates (DESEQ2). (b) MAplot (DESEQ2). Each red dot represent a differentially expressed gene (p<0.0001) while grey dots represents non differentually expressed genes.At least 87% of trimmed reads were uniquely mapped in Rice nipponbare genome.

FIGURE S5

A

SampleID	Clean_Reads	GC(%)	N(%)	Q30(%)
pine1 rep1 AC	22,440,398	49.03	0	97.69
pine1 rep1 I	32,101,162	44.58	0	98.21
pine1 rep2 AC	21,502,771	49.76	0	97.63
pine1 rep2 I	31,118,646	44.77	0	98.15
WT rep1 AC	25,745,153	49.59	0	97.58
WT rep1 I	37,516,283	44.29	0	98.16
WT rep2 AC	26,937,311	48.24	0	97.60
WT rep2 I	44,068,722	52.16	0	97.49

B

SampleID	Total_Reads	Mapped_Reads	Uniq_Mapped_Reads	Duplication(%)
WT rep1 AC	25,745,153	24,848,721(96.52%)	20,867,285(81.05%)	13.82
WT rep1 I	37,516,283	36,797,668(98.08%)	28,880,088(76.98%)	6.68
WT rep2 AC	26,937,311	26,272,313(97.53%)	21,235,681(78.83%)	14.21
WT rep2 I	44,068,722	43,050,737(97.69%)	31,731,091(72%%)	7.01
pine1 rep1 AC	22,440,398	21,744,272(96.90%)	18,098,768(80.65%)	12.63
pine1 rep1 I	32,101,162	31,568,621(98.34%)	24,811,528(77.29%)	5.71
pine1 rep2 AC	21,502,771	20,804,494(96.75%)	17,434,467(81.08%)	12.43
pine1 rep2 I	31,118,646	30,596,983(98.32%)	23,984,951(77.07%)	6.32

C

SampleID	Number	Min_length	1st_quarter_length	Median	Mean	3rd_quarter_length	Max_length
WT rep1 AC	40,414	150	507	720	785	971	3,492
WT rep1 I	9,259	150	176	214	298	282	4,699
WT rep2 AC	41,646	150	451	674	735	922	5,727
WT rep2 I	170,516	150	326	594	700	942	4,552
pine1 rep1 AC	40,159	150	470	680	745	930	4,236
pine1 rep1 I	9,034	150	174	209	283	274	4,677
pine1 rep2 AC	40,897	150	504	715	783	979	4,325
pine1 rep2 I	8,825	150	175	214	290	284	4,669

D

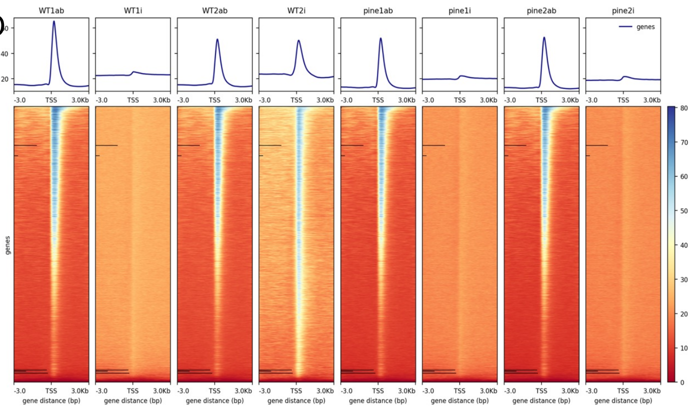

E

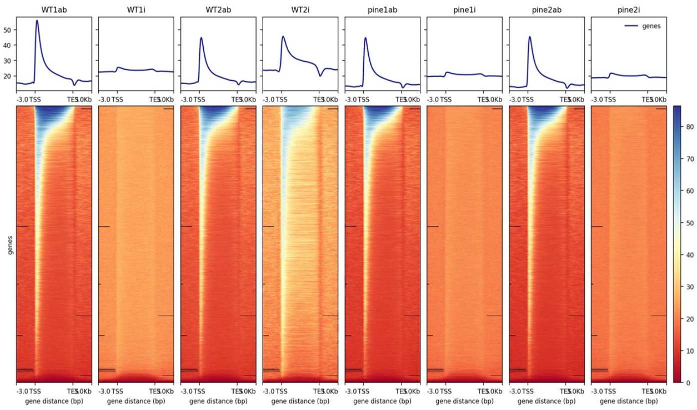

Fig.S5. ChIP-Seq data quality (a) Clean statistics: SamplesID: Sample name; Clean_Reads: Counts of clean PE reads; GC(%): Percentage of G, C in clean data; N(%): Percentage of N in clean data; ≥Q30%: Percentage of bases with Q-score no less than Q30. (b) Mapping statistics: Sample_id: Sample ID in the system; Total_Reads: Counts of clean reads, as the single end; Mapped_Reads: Counts of mapped reads and the proportion of that in clean data; Uniq_Mapped_Reads: Counts of reads mapped to a unique position on the reference genome and proportion of that in clean data; MT(%): Percentage of mitochondrial reads in clean reads; Duplication(%): Percentage of duplication reads in all clean reads. (c) Statistics on genome-wide peak calling.(d) Number of the total reads around the TSS and (e) from the TSS to the TES in the samples subjected to ChIP-seq. i: input; ab: IP. DeepTools was used to draw the density distribution map of the sequencing reads between the upstream and downstream 3 for each gene, and the results were presented in the form of heatmaps.

FIGURE S6

A

SampleID	Raw_Reads	Clean_Reads	GC(%)	N(%)	Q30(%)
pine1	31,750,556	31,732,930	46.12	0	94.95
pine2	35,239,710	35,221,307	46.27	0	94.99
pine3	35,083,418	35,064,767	45.73	0	94.87
pine4	40,518,690	40,493,739	46.30	0	95.00
wt1	35,916,415	35,895,700	46.02	0	95.00
wt2	35,900,441	35,879,963	46.03	0	95.34
wt3	34,468,350	34,441,985	46.88	0	95.42
wt4	36,013,669	35,993,042	45.86	0	95.03

B

SampleID	Total_Reads	Mapped_Reads	Uniq_Mapped_Reads	MT(%)	Duplication(%)
pine1	31,732,930	29,592,790(93.26%)	17,980,883(56.66%)	0	11.04
pine2	35,221,307	33,046,896(93.83%)	20,377,087(57.85%)	0	10.41
pine3	35,064,767	32,698,871(93.25%)	18,756,911(53.49%)	0	10.31
pine4	40,493,739	37,908,846(93.62%)	23,525,842(58.09%)	0	10.83
wt1	35,895,700	33,296,786(92.76%)	20,953,548(58.37%)	0	11.50
wt2	35,879,963	33,578,732(93.59%)	21,579,257(60.14%)	0	11.14
wt3	34,441,985	32,100,519(93.20%)	20,749,375(60.24%)	0	9.79
wt4	35,993,042	32,985,259(91.64%)	19,993,626(55.54%)	0	10.86

C

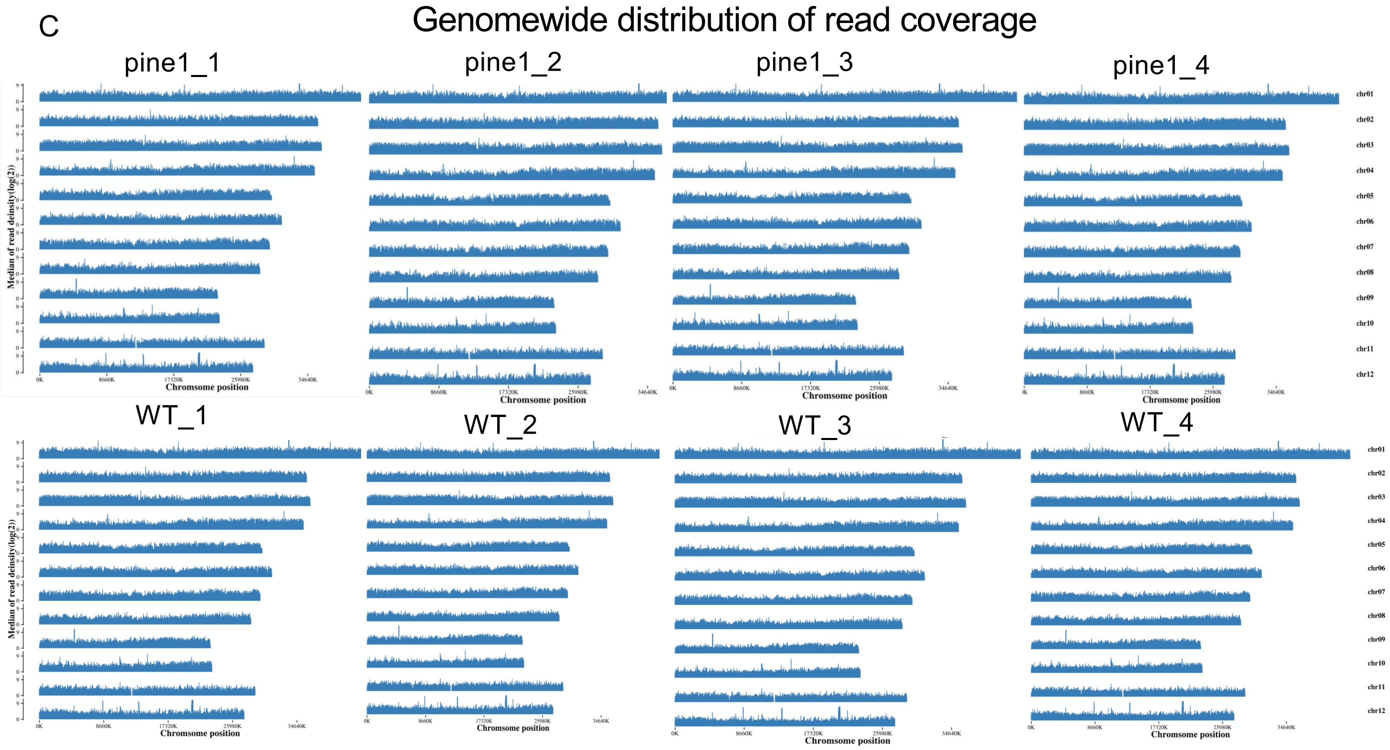

D

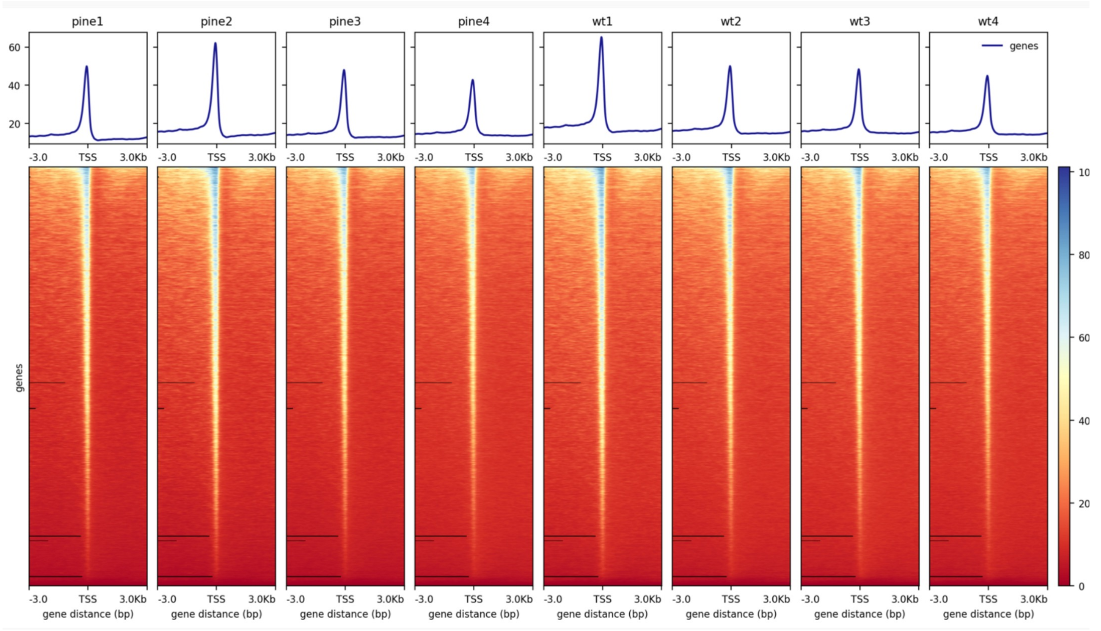

E

SampleID	Number	Min_length	1st_quarter_ length	Median	Mean	3rd_quarter_ length	Max_length
pine1	84,237	150	246	362	433	545	3,578
pine2	92,360	150	246	365	440	556	3,581
pine3	80,269	150	245	366	441	557	4,675
pine4	77,027	150	241	362	436	548	4,673
wt1	89,876	150	248	368	444	558	4,674
wt2	83,732	150	246	372	446	566	3,581
wt3	83,234	150	235	351	424	537	3,575
wt4	76,953	150	239	358	432	545	3,582

F

SampleID	Promoter (<=1kb)(%)	Promoter (1-2kb)(%)	Promoter (2-3kb)(%)	3' UTR(%)	5' UTR(%)	Exon(%)	Intron(%)	Distal Intergenic(%)
pine1	35.20	16.36	11.64	2.34	0.19	0.64	0.87	32.76
pine2	34.52	16.70	11.84	2.67	0.19	0.65	0.84	32.60
pine3	36.26	16.28	11.55	2.37	0.17	0.67	0.86	31.83
pine4	37.29	16.08	11.27	2.11	0.19	0.67	0.87	31.52
wt1	34.99	16.52	11.80	2.43	0.19	0.68	0.87	32.52
wt2	35.88	16.43	11.65	2.19	0.19	0.61	0.86	32.21
wt3	36.98	16.28	11.36	1.91	0.17	0.67	0.87	31.75
wt4	36.91	16.22	11.44	1.93	0.18	0.58	0.87	31.87

G

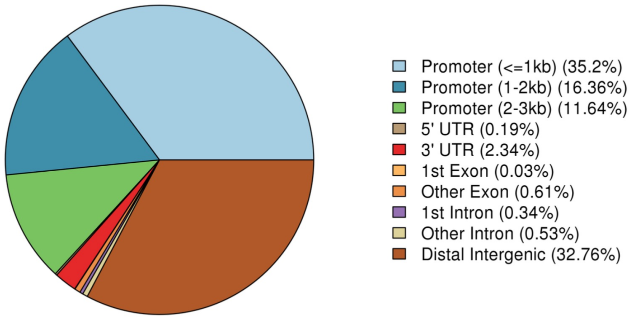

Fig.S6. ATAC-Seq data quality (a) Clean data statistics SamplesID: Sample name; Raw_Reads: Counts of raw PE reads; Clean_Reads: Counts of clean PE reads; GC(%): Percentage of G, C in clean data; N(%): Percentage of N in clean data; ≥Q30%: Percentage of bases with Q-score no less than Q30. (b) Mapping statistics: Sample_id: Sample ID in the system; Total_Reads: Counts of clean reads, as the single end; Mapped_Reads: Counts of mapped reads and the proportion of that in clean data; Uniq_Mapped_Reads: Counts of reads mapped to a unique position on the reference genome and proportion of that in clean data; MT(%): Percentage of mitochondrial reads in clean reads; Duplication(%): Percentage of duplication reads in all clean reads. (c) Whole genome coverage distribution map. X-axis: Position on the chromosome; Y-axis: Log2 of coverage depth (coverage depth was defined as reads counted within a chromosome window of 10 kb in length). (d) Evaluation of Enrichment of Sequencing Data Near TSS DeepTools 3.2.0was used to draw the density distribution map of the sequencing reads between the 3 kb of each upstream and downstream of TSS for each gene, and the results were presented in the form of heatmaps. (e) Statistics on genome-wide peak calling. (f, g) Statistics on the distribution of peak in different gene functional elements.

FIGURE S7

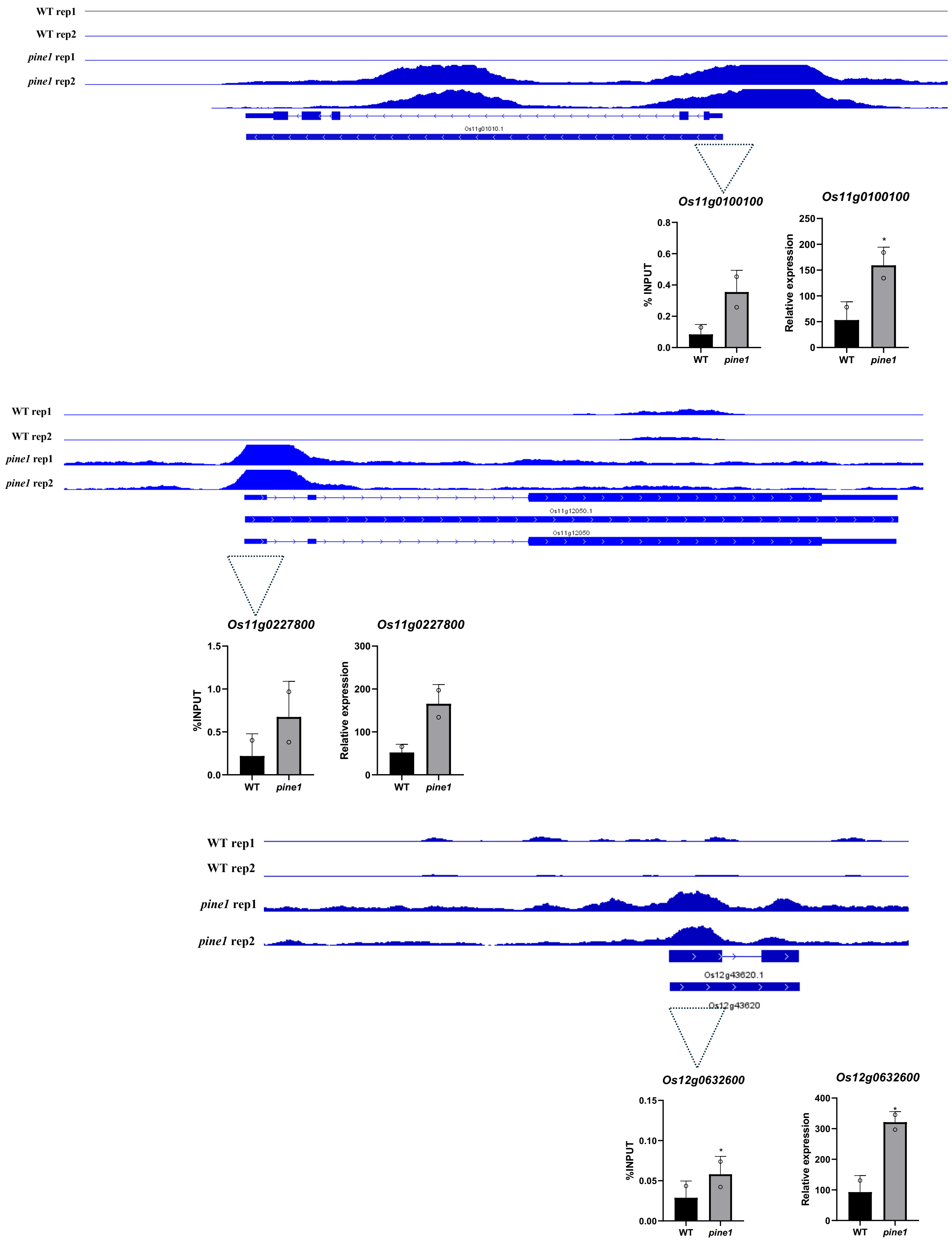

Fig.S7. **Dataset validation**. Acetylation pattern in *wt* and *pine1* samples and gene structure. Inverted triangles indicate the position where primers forChIP -qPCR and qRT-PCR where designed in the three genes, belonging to the overlap between Chip-seq, RNA-seq and ATAC-seq datasets. Gene expression is relative to UBQ.

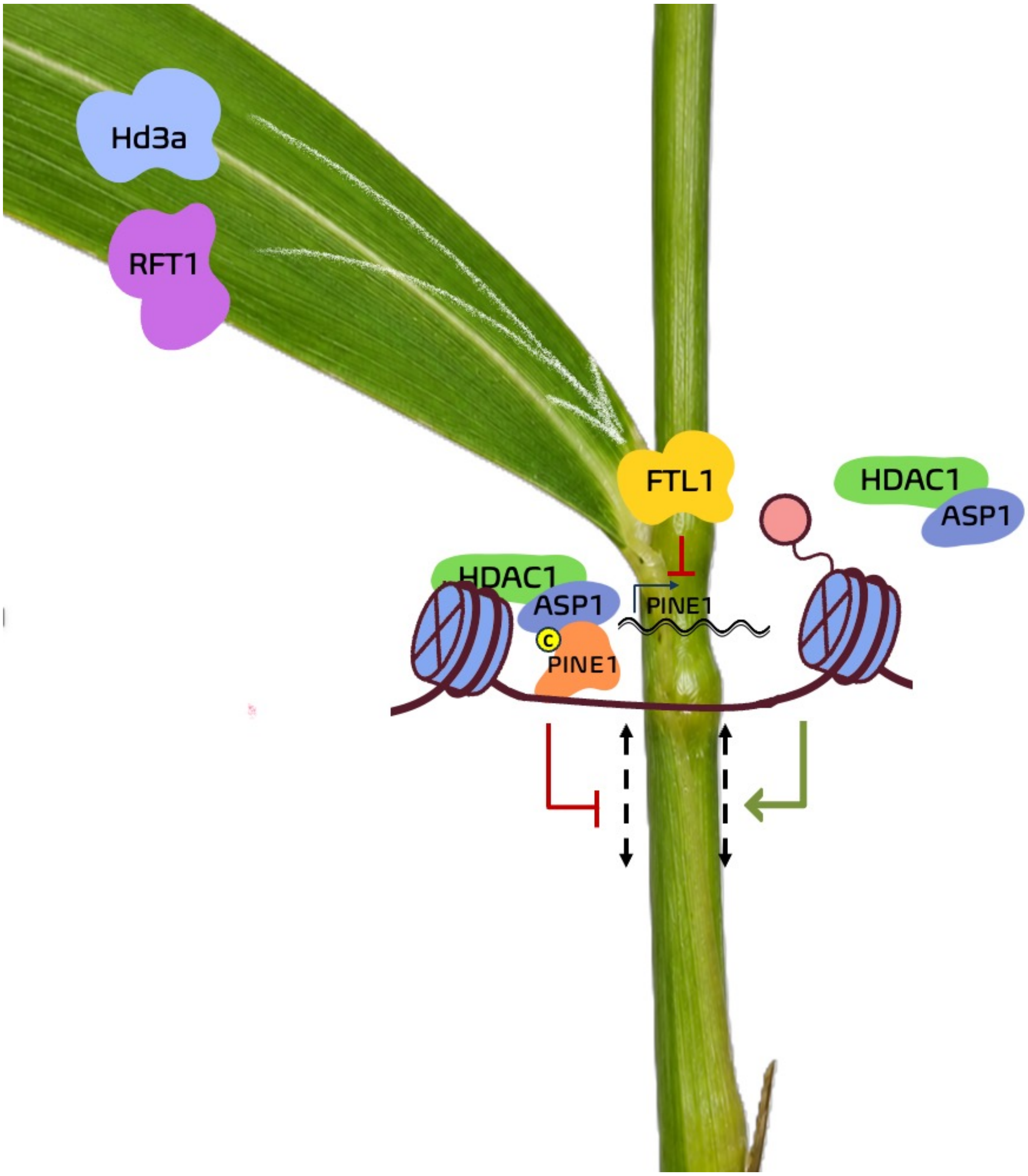

Fig.S8. Working model, integrating the position of *PINE1* transcriptionally regulated by OsFT-L1, in the florigens regulatory network and its molecular function.
